# The Computational Studies of Plasmon Interaction

**DOI:** 10.1186/s11671-017-2050-8

**Published:** 2017-04-13

**Authors:** Antonina Demchuk, Ivan Bolesta, Oleksii Kushnir, Ihor Kolych

**Affiliations:** Department of Radiophysics and Computer Technologies, Ivan Franko National University of Lviv, Generala Tarnavskoho Str. 107, Lviv, 79017 Ukraine

**Keywords:** Nanoparticle, Surface plasmon resonance, Optical spectra, Extinction cross section, Hybridization model, Dimer, Fractal cluster, Discrete dipole approximation

## Abstract

In this paper, an interaction of metal nanoparticles that appears in the extinction spectra was investigated. The mutual coupling between the nanoparticles, the effect of size difference, and the interparticle separation in silver nanoparticle dimers are studied by computer discrete dipole approximation methods. The obtained results show that nanoparticle interaction results in the distinct collective modes, known as the low-energy bonding modes and the higher-energy antibounding modes. The spectral position of the modes is analyzed as a function of the ratio of interparticle distance to particle size that reduces the dependency on the particle size itself. The optical spectra of nanoparticles that form the fractal cluster were investigated. It was found that the number of spectral bands increase with the growth of the number of nanoparticles in the fractal cluster, which are described within the plasmon hybridization model.

## Background

Metal nanoparticles have attracted a great attention due to their strong interaction with light. The interesting optical properties of metal nanoparticles, such as bright intense colors, are the result of interaction of free carriers with the incident electric field. In the presence of the oscillating electromagnetic field of the light, the free electrons of the metal nanoparticle undergo a collective coherent oscillation with respect to the positive metallic lattice [1]. This process is resonant at a particular frequency of the light and is called the localized surface plasmon resonance (LSPR) oscillation.

In a single metal nanoparticle, frequency, strength, and quality of the LSPR depends on the size, geometry, the metal composition, and the refractive index of the local environment. Furthermore, the LSPR of a metal nanoparticle is sensitive to the presence of other nearby metal nanoparticles, their sizes, interparticle distance, and materials. In an assembly of metal nanoparticles with small interparticle distance compared to the size of particle, the LSPR is strongly affected by the near-field coupling of the individual particles [2].

Another aspect of the study is the plasmon spectra of metal nanoparticles associated with the formation of metal-dielectric nanocomposites. Nanocomposites can cause significant influence on linear and nonlinear susceptibilities of matrix [3], radiation recombination processes [4], and giant surface-enhanced Raman scattering (SERS)[5]. Practical interest in dielectric materials with metal nanoparticles is connected with perspective for development of optical switches on their basis with ultra-short time response, limiters of optical laser beam intensity, for synchronization of the laser modes, etc.

Progress in the investigation of plasmonic spectra of the metal nanoparticles has been greatly assisted by developments in technology, experimental techniques, and numerical modeling of their extinction spectra. This allows to study the impact of different factors on the extinction spectra of nanoparticles and their composites.

Pairs of nanoparticles called “dimers” are the simplest model systems to study as only two directions of incident polarization required to explore the interparticle coupling completely. It is shown [6] that dimers with an interparticle distance smaller than the diameter of the nanoparticles can generate high plasmonic enhancements.

The optical spectra of individual particle, dimers and the chains consisting of particles of the same size, the dependence on the medium host, the interparticle separation, and the direction of incident polarization were studied in [7]. The authors of [8] explored the extinction spectra and plasmon hybridization schemes of nanoparticle dimers of various sizes and materials. In [9], the field enhancement spectra were investigated as a function of the particle size difference and the interparticle spacing, and also, localized surface plasmon resonances in the chains of different particle sizes were analyzed depending on the particle size and interparticle separation [10].

The general case of the spherical nanoparticle coupling is fractal clusters. Since fractal clusters are characterized by local anisotropy of each particle surroundings, the supplements compensation to local field (including the dominant surroundings contribution) do not occur. This leads to the emergence of strong local electromagnetic fields, which will vary in different parts of fractal cluster (field fluctuation)[11]. Local fields, in turn, enhance the optical susceptibilities of particles that reflects in the linear and nonlinear optical characteristics of fractal clusters [12]. As a result, the extinction spectra of fractal clusters differ significantly from the spectra of individual material nanoparticles.

This paper studies the nanoparticle interaction that shows up in the extinction spectra. The spectra of the simple nanoparticle system (dimers with particles of similar and dissimilar sizes) were analyzed. The changes of spectra are investigated to depend on the interparticle distance and the interaction of particles as the part of fractal cluster.

## Methods

We use the discrete dipole approximation (DDA) method to simulate the distribution of the electromagnetic field for the composition of silver nanoparticles. Extinction cross sections are calculated from the resulting polarizations of DDA method, using the solving package “EMSimulation” [13]. The dielectric function of silver nanoparticles is taken as a bulk silver from the Johnson and Christy table [14] with phenomenological correction for size reduction effects. The particles are assumed to be embedded in a vacuum, which is described by a relative permittivity *ε*=1 or in dielectric media with *ε*=*n* ^2^, where *n* is the refractive index.

The DDA is a method for calculating scattering and absorption of electromagnetic waves by nanocomposites. The DDA was proposed in 1973 by Purcell and Pennypacker [15], who used it to study interstellar dust grains. The main idea of the method is that the target is approximated by an array of *N* point dipoles at positions *r*_*i*_ with polarizabilities *a*_*i*_. The polarization

1 $$ P_{i}=a_{i} \cdot E(r_{i})  $$

of each dipole responds to the total electric field at its position; *E*(*r*_*i*_) is the sum of an incident plane wave

2 $$ E_{\text{inc},i}=E_{0}\exp(ik \cdot r_{i} - i \omega t)  $$

and a contribution from all the other dipoles

3 $$ E_{\text{other},i}=- \sum_{i \neq j} A_{ij} \cdot P_{j}.  $$

The medium of the target is characterized by its complex dielectric function *ε*. The dipole polarizabilities *a*_*i*_ can be given by the Clausius-Mosotti polarizability [16]:

4 $$ a_{i} = \frac{3d^{3}}{4 \pi} \left(\frac{\varepsilon_{i}^{2} + 1}{\varepsilon_{i}^{2} + 2}\right),  $$

where *d* is the diameter of the dipole. Draine [17] showed that for finite wavelengths, the optical theorem requires that polarizabilities include also the radiative-reaction correction of the form

5 $$ \alpha=\frac{\alpha^{(nr)}}{1 - (2/3) i (\alpha^{(nr)} / d^{3}) (kd)^{3}},  $$

where *α* ^(*nr*)^ is the nonradiative polarizability.

The DDA method allows to get an oscillating dipole moments *P*_*j*_ for every monochromatic incident wave; from these *P*_*j*_ the absorption and scattering cross sections are computed [18]:

6 $$ C_{ext}=\frac{4 \pi k}{{\left| E_{0} \right|}^{2}} \sum_{j=1}^{N} Im\left(E_{inc,j}^{*} \cdot P_{j}\right)  $$

7 $$ C_{abs}=\frac{4 \pi k}{{\left|E_{0}\right|}^{2}} \sum_{j=1}^{N} \left\{Im\left(P_{j} \cdot \left(\alpha_{j}^{-1}\right)^{*} P_{j}^{*}\right) - \frac{2}{3}k^{3} {\left| P_{j} \right|}^{2} \right\}  $$

To compare cross sections of dipoles with different radiuses, we use effective cross sections, which are calculated as

8 $$\begin{array}{*{20}l} Q_{\text{ext}}&=C_{\text{ext}}/A, \end{array} $$

9 $$\begin{array}{*{20}l} Q_{\text{abs}}&=C_{\text{abs}}/A, \end{array} $$

where *A* is the area of overlap between the incident beam and the target object.

The scattering problem for the array of point dipoles, represented by the system of linear equation, can be solved with arbitrary accuracy. We use the fast Fourier transform (FFT) techniques together with the conjugate gradient method to obtain solutions for targets.

We calculated extinction spectra of fractal clusters with increasing degree of aggregation of nanoparticles in the clusters. Simulations of fractal clusters growth conducted in a “cluster-cluster” model [19].

To decompose the optical spectra into peak functions, we performed two steps. Firstly, the method of second derivative spectroscopy [20] was used to find the hidden peaks for the initial definition of the peak position. Then, L-BFGS (Broyden-Fletcher-Goldfarb-Shanno) [21] algorithm was used to reduce the standard deviation between the peak superposition and the simulated spectrum of fractal cluster.

## Results and Discussion

**Fig. 1:**
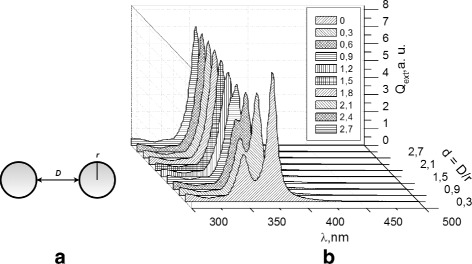
**a** The dimer with nanoparticle radius *r* and interparticle distance *D*. **b** The effective extinction spectra of nanoparticle dimer dependent on the interparticle separation *d*

Calculation of extinction spectra of dipoles shows that the interaction between the nanoparticles, which depends on the distance between them, manifested in the appearance of two components (Fig. 1). Spectral distance *Δ* *λ* between components depends on the distance (*D*) between nanoparticles (for fixed radius *r*), on the size of nanoparticles (for fixed distances), and on the permittivity of the environment.

In the case of dielectric medium other than vacuum, the behavior of the spectra remains qualitatively the same, but the band shifted to long wavelength region (for isolated particles and dimers) and splitting between the bands increases. Analysis of the results shows that the ratio *d*=*D*/*r* is more universal because it minimizes the dependency on the nanoparticle radius *r*.

Figure 1 shows effective cross section extinction spectra of dimer in vacuum with similar size particles with radius *r*=10 nm dependent on interparticle separation *d*, where *d*=*D*/*r* depends on the radius of a nanoparticle *r* and the interparticle distance *D* (Fig. 1 a). Cross section extinction was calculated under the longitudinal, transverse, and oblique incident field polarization. Both the longitudinal and transverse plasmon coupling bands for the homodimer can be recognized under the oblique polarization.

Calculation results show that at the interparticle separation *d*>2, only one strong plasmon coupling absorption band can be observed. After decreasing the interparticle distance, the single plasmon resonance band is split into two plasmon bands forming two different peaks (Fig. 1 b).

When the gap distance between nanoparticles is large enough, every metal nanoparticle exists as an individual, so the interaction between nanoparticles is very weak. When the gap is quite small, the system of nanoparticles consider to be as a whole. So in both cases, it is impossible to cause the energy splitting and the resonance peak position shifting.

Moreover, the plasmon coupling of a dimer shows a large red-shift as the interparticle distance decreases for incident polarization parallel to the dimer axis, due to the applied and induced electric fields that are added to each other. For incident polarization perpendicular to the axis, a destructive interaction between the applied and induced electric fields is predicted, leading to a small blue spectral shift [22].

**Fig. 2:**
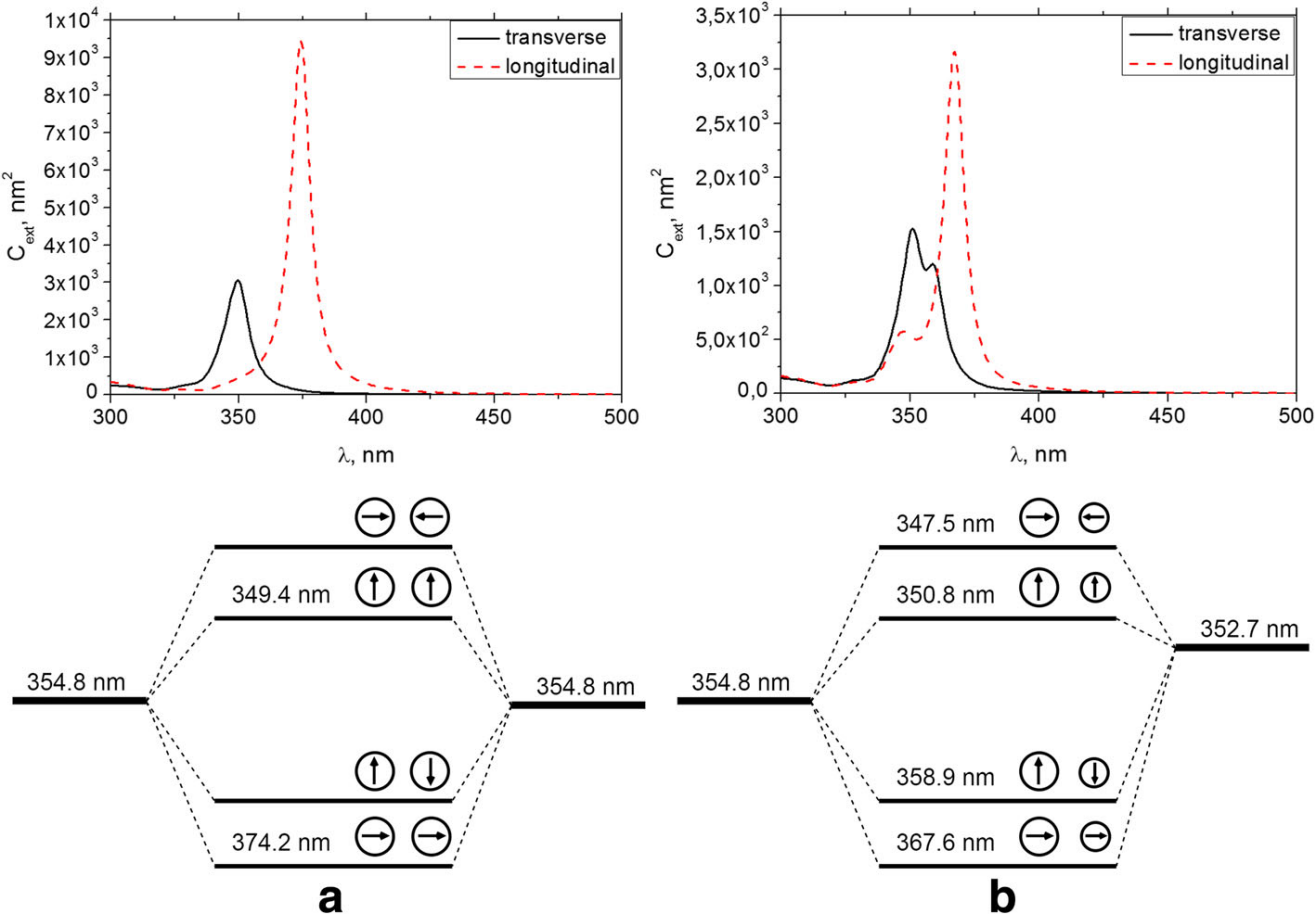
The extinction spectra of the dimer under the transverse and longitudinal incident field polarization and the plasmon hybridization schema for **a** dimer with the same nanoparticle size *r*=10 nm (homodimer), **b** dimer with different nanoparticle sizes: *r*_1_=10 nm and *r*_2_=4 nm (heterodimer)

The extinction spectrum of a symmetric dimer displays two distinct modes (Fig. 2 a), the lower energy one at 374.2 nm resulting from the longitudinal coupling of the particle plasmons and the higher-energy one at 349.7 nm from the transverse coupling.

This behavior in electromagnetic coupling of the plasmon resonance between two nanoparticles can be explained by the phenomenon of molecular hybridization. This means when very strong plasmonic enhancement is in place within a dimer, two plasmons hybridize to form a lower energy bonding plasmon mode and a higher-energy antibonding plasmon mode [23].

In order to demonstrate the effect of introducing a size asymmetry in the coupling scheme, we discuss the LSPR scattering spectra obtained from an asymmetric dimer composed of a 4- and 10-nm silver particle.

The extinction spectra were calculated (Fig. 2 b) for two extreme orthogonal polarizations of the asymmetric dimer, each contains two modes at 350.8 and 358.9 nm for the transverse and at 347.5 and 367.6 nm for the longitudinal polarization, respectively. This observation is in direct contrast to the homodimer case (Fig. 2 a) and can be understood by introducing the asymmetry in the plasmon hybridization model, as depicted in the hybridization scheme.

When the sizes of nanoparticles in the system are different, the electric field can no longer be assumed to be uniform inside the particles, and high-order (quadrupole, octupole, etc.) plasmon modes can directly couple with the electric field of the light simply due to the phase retardation effect. Excitation of multipole plasmon resonances is the result of the asymmetric distribution of the surface plasmons caused by the electromagnetic interactions between the localized modes. This effect may be used to enhance the nonlinear optical response of an effective medium composed of particles with engineered size dispersion and particle placement [10].

**Fig. 3:**
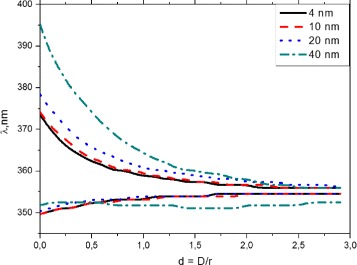
The longitudinal and transverse field enhancement peaks dependent on the interparticle separation for dimers with radiuses *r*=4 nm, *r*=10 nm, *r*=20 nm, and *r*=40 nm

Figure 3 shows relation of the longitudinal and the transverse field enhancement peak to interparticle separation for different radiuses of nanoparticles in the homodimer. The figure shows the exponential relationship between the position of the peak enhancement and the interparticle distance of dimer with the asymptotical approaching the wavelength value 354.8 nm that corresponds to the isolated sphere enhancement.

The spectral distance *Δ* *λ* between the enhancement peaks follows a universal scaling law as a function of the dimensionless parameter *d*=*D*/*r* (quasi-exponential behavior) and has given birth to the concept of "plasmon ruler" that may be used to infer the length of a molecular chain connecting functionalized nanoparticles.

**Fig. 4:**
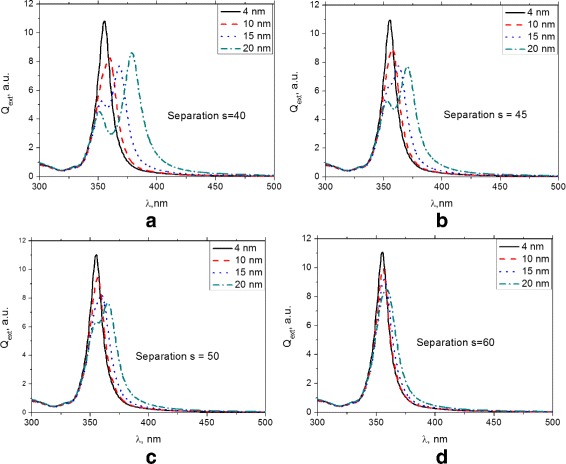
The internal field enhancement spectra for four silver nanoparticle dimers with different radius ratios, at a fixed center-to-center separation **a** *s*=40 nm, **b** *s*=45 nm, **c** *s*=50 nm, and **d** *s*=60 nm. The radius of the small nanoparticle is varied while that of the large nanoparticle is kept constant at 20 nm

Figure 4 a show the results for a dimer with a different radius ratio. The radius of the small nanoparticle is varied while that of the large nanoparticle is kept constant at 20 nm. The field enhancement shows two resonance peaks for the lower radius ratio. At a larger volume ratio, the peak field enhancement has increased. With increase in interparticle spacing, the peak enhancement shifts to lower wavelengths and shows less pronounced peak splitting. The smaller separation leads to an increased field enhancement due to the larger near field provided by the bigger size particle at this distance.

For small size difference or large interparticle spacing, low-field enhancement peaks are observed. For most geometries, the field enhancement peak exceeds that of an isolated silver nanoparticle, with the exception of dimers with small radius ratio or large interparticle spacing. The largest field enhancement peaks are observed at small spacing and large size difference.

**Fig. 5:**
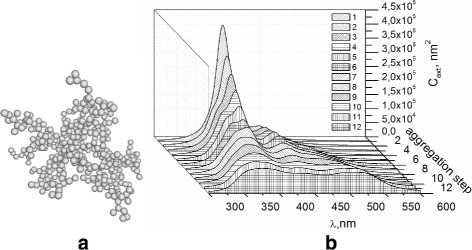
**a** The fractal cluster simulated within the model of “cluster-cluster.” **b** Extinction spectra of the nanoparticles at different stages (curves 1–12) of fractal cluster formation

We calculated the extinction spectra of nanoparticles which form the fractal cluster. Simulation of fractal cluster growth was carried out from 1000 polydisperse nanoparticles within the model of “cluster-cluster” (Fig. 5 a), which corresponds to the natural growth of fractal aggregates in sols. The radius of nanoparticles is normally distributed with the mean of 5 nm and the dispersion of 1 nm.

Figure 5 b presents the change (dynamics) of the extinction cross section spectra within the fractal grows stages. Curve 1 shows the spectrum of non-interacting particles, curves 2—11 show the spectra of clusters, with increasing number of particles respectively, curve 12 shows the spectrum of fractal clusters with all joined nanoparticles.

**Fig. 6:**
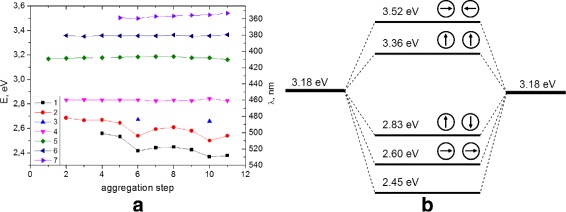
**a** The spectral position of components at different stages of cluster formation. The numbers on the horizontal axis correspond to the numbers of curves in Fig. 5. **b** The hybridization scheme of plasmon modes in fractal cluster

Analysis of the spectra by derivative spectroscopy method revealed the number of spectral components (peak functions) in the cluster (Fig. 6 a). It is shown that the number of spectral components increase with the growth of the number of nanoparticles in the fractal cluster at different stages of cluster formation.

On the spectrum of non-interacting nanoparticles (aggregation step 1), one band with a maximum at 3.18 eV can be observed (curve 5 at Fig. 6 a). The spectra of the initial stages of cluster formation (aggregation steps 2 and 3) show the additional three bands with the maximum at 2.60, 2.83, and 3.36 eV (curves 2, 4, and 6 in Fig. 6 a). At the later stages of aggregation, the additional bands at 2.45 and 3.52 eV are present (curves 1 and 7 in Fig. 6 a).

Figure 6 a shows that the spectral position of the component in spectra does not depend on the degree of aggregation. For most components, the band energy does not change significantly at different stages of cluster formation. For the low-energy components (curves 1 and 2 in Fig. 6 a), we can observe large red-shift with the growth of cluster, and for the high-energy components (curve 6, 7 in Fig. 6 a), small blue-shift is present. This phenomenon can be explained by interaction of large number of nanoparticles in cluster and corresponds to the previously obtained results for the simple system of nanoparticle dimer.

The difference between the extinction spectra of fractal clusters and non-interacting spectra is shown on the hybridization scheme on Fig. 6 b. Low-energy bands at 2.60 and 2.83 eV can be associated with bounding orbitals corresponding to different orientations of interacting dipoles. High-energy bands at 3.36 and 3.52 eV are associated with loosening plasmon modes. Moreover, the most high-energy mode appears in clusters with sufficiently large number of particles (curve 7 at Fig. 6 a).

Dipole-dipole interaction of nanoparticles can cause splitting in the energy modes, as shown in hybridization schema (Fig. 6 b). The low energy band at 2.45 eV does not correspond to dipole mode interaction and can appear at higher levels of aggregation, as a result of high-order modes interaction (quadrupole, octupole, etc.).

## Conclusions

In summary, the optical properties of plasmon resonant metal nanocomposites were investigated. Plasmon resonances are numerically evaluated in the silver nanosphere dimers of similar and different sizes and in the fractal cluster containing silver nanoparticles embedded in a vacuum.

We have shown that the interparticle coupling interaction among the random distribution of silver nanoparticles can lead to hybridization, splitting, and shifting of the plasmon energies, as well as the quadrupolar resonances formed by the interaction between the plasmons of the metal nanoparticles.

The extinction spectra in dimers with identical nanoparticle sizes indicate the presence of a dipolar particle response. However, the interaction of particles with dissimilar sizes also results in the splitting of the plasmon resonances into two resonances: the lower energy bounding plasmon and the higher-energy antibounding plasmon.

The change of the maximum band as a function of the particle size and interparticle separation was analyzed. It is shown that the maximum band depends on the ratio of the interparticle distance to the size of particles, and this relation is more universal because it minimizes the dependency on the nanoparticle size.

In the fractal cluster consisting of similar nanoparticles the additional energy modes can appear that indicates the presence of higher-order plasmon modes (quadrupole or higher). The spectral position of maximum bands does not change significantly with the degree of aggregation of cluster. But the higher modes mostly appears on the later stages of fractal cluster formation.

The obtained results can be used for analyzing the real nanocomposites, determining the interparticle distance between nanoparticles, and creating the nanomaterials with special properties.

## Abbreviations

DDA: Discrete dipole approximation
FFT: Fast-fourier transform
L-BFGS: Limited-memory Broyden-Fletcher-Goldfarb-Shanno
LSPR: Localized surface plasmon resonance
SERS: Surface-enhanced Raman scattering
